# Examining dose-response of an outdoor walk group program in the Getting Older Adults Outdoors (GO-OUT) trial

**DOI:** 10.1371/journal.pone.0309933

**Published:** 2025-03-13

**Authors:** Tai-Te Su, Ruth Barclay, Rahim Moineddin, Nancy M. Salbach

**Affiliations:** 1 Department of Physical Therapy, University of Toronto, Toronto, Ontario, Canada; 2 Department of Physical Therapy, University of Manitoba, Winnipeg, Manitoba, Canada; 3 Department of Family and Community Medicine, University of Toronto, Toronto, Ontario, Canada; 4 KITE Research Institute, Toronto Rehabilitation Institute—University Health Network, Toronto, Ontario, Canada; University of Pavia, ITALY

## Abstract

**Objective:**

The Getting Older Adults Outdoors (GO-OUT) randomized trial showed that a 10-week outdoor walk group (OWG) program was not superior to 10 weekly phone reminders in increasing physical and mental health; however, OWG attendance varied. This study examined whether dose-response relationships existed between OWG attendance and improvement in physical and mental health among older adults with mobility limitations.

**Methods:**

We analyzed data from 76 OWG participants with pre- and post-intervention scores on at least one of seven measures of health outcomes (walking endurance, comfortable and fast walking speed, balance, lower extremity strength, walking self-efficacy, and emotional well-being). Participants were classified as attending 0–9, 10–15, and 16–20 OWG sessions based on attendance tertiles. We adjusted for participant sex and study site in regression analyses.

**Results:**

Among the 76 participants, mean age was 74.9 ± 6.6 years and 72% were female. Compared to those attending 0–9 OWG sessions, participants attending 16–20 sessions exhibited a 56.3-meter greater improvement in walking endurance (95% CI: 17.3, 95.4, *p* = 0.005); 0.15-meter/second greater improvement in comfortable walking speed (95% CI: 0.01, 0.29, *p* = 0.034); and 0.18-meter/second greater improvement in fast walking speed (95% CI: 0.03, 0.34, *p* = 0.020). Higher attendance was associated with greater odds of improvement in comfortable walking speed (OR = 7.1; 95% CI: 1.1, 57.8, *p* = 0.047) and fast walking speed (OR = 10.1, 95% CI: 1.8, 72.0, *p* = 0.014). No significant dose-response relationships for the remaining outcomes were observed.

**Conclusions:**

Higher attendance in a park-based, supervised, task-oriented and progressive OWG program is associated with greater improvement in walking endurance and walking speed among older adults with mobility limitations. Attendance likely impacted walking capacity and not balance, lower extremity strength, walking self-efficacy or emotional well-being due to task-specificity of training. This study highlights the importance of attendance when designing and implementing OWG programs to enhance walking endurance and speed among older adults.

## Introduction

Walking in outdoor natural environments plays a critical role in supporting the health and well-being of older adults. An accumulating body of evidence has shown that outdoor walking is associated with a wide array of health benefits. These benefits include improved physical activity and fitness [1,2], better cognitive and mental functioning [3,4], increased energy and greater feelings of revitalization [5]. In addition, exposure to natural environments has been linked to a decreased incidence of chronic health conditions such as diabetes, stroke, coronary heart disease, and all-cause mortality [6,7]. The combination of physical activity and natural environments makes outdoor walking an ideal and promising strategy for health promotion among older adults.

Although walking outdoors is a preferred mode of physical activity [5], a recent systematic review and meta-analysis found no significant difference between outdoor community ambulation and other comparison interventions (e.g., standard care or educational lectures) in improving older adults’ walking endurance and depression [8]. While the limited number of studies included and quality of evidence partly account for this phenomenon [8], attendance may play a major role in shaping the overall effects of outdoor walking. In particular, previous studies have shown that attendance, defined as the number or proportion of sessions attended, can fluctuate between 58% and 77% among older adults participating in exercise interventions [9–14]. Attendance in exercise programs is of special importance for older adults with mobility limitations, as research indicates that poor attendance or non-attendance are significant risk factors for adverse outcomes such as more frequent falls and increased utilization of healthcare resources [9,15]. Given that attendance levels can affect the total amount of training and environmental stimuli received, it is important to understand whether and how the effects of outdoor walking may vary depending on participants’ attendance to the interventions.

The Getting Older Adults Outdoors (GO-OUT) study [16] provides a basis for examining the impact of outdoor walking attendance on older adults’ health. The GO-OUT trial is a randomized controlled trial designed to evaluate the effects of a park-based, task-oriented walking program on walking activity and capacity among older adults with mobility limitations [16]. One group participated in a 1-day educational workshop plus a 10-week outdoor walk group (OWG) program, while the other group completed the same workshop and received a weekly telephone reminder for 10 weeks. Despite participants providing positive feedback on the OWG program [17], our quantitative analyses revealed no significant difference between the two groups in changes in minutes walked outdoors; health promoting behaviors, or successful aging pre- to post-intervention [18]. The OWG only led to greater improvement in walking capacity through walking self-efficacy compared to the weekly reminders group [18]. As noted in the process evaluation of the GO-OUT trial [19], the observed lack of effects may be attributed to varying levels of attendance within the OWG, as mean attendance ranged from 43.8% to 84.9% across the study sites.

To better understand the benefits of outdoor walking interventions and inform their implementation in community settings, the objective of this study was to explore whether a dose-response relationship existed between attendance in the OWG program and improvement in physical and mental health among older adults with mobility limitations. We focused on seven physical and mental health outcomes measured in the GO-OUT trial: walking endurance, comfortable and fast walking speed, balance, lower extremity strength, walking self-efficacy, and emotional well-being. These outcomes were investigated as they are essential for promoting healthy aging and improving health-related quality of life in older adult populations, making them key indicators for evaluating the benefits of the OWG program [17,20,21]. Knowledge derived from the present study will not only help to equip clinical practitioners with evidence-based recommendations but also inform marketing strategies to motivate older adults to engage with community-based exercise programs.

## Methods

### Study design

The GO-OUT randomized controlled trial was conducted in four urban cities in Canada (Edmonton, Winnipeg, Toronto and Montreal), and was registered on ClinicalTrials.gov (registration number: NCT03292510). A detailed study protocol for the GO-OUT trial has been reported [16]. Between February 20, 2018 and May 15, 2019, the GO-OUT trial enrolled 190 older adults with mobility limitations. Upon enrollment, participants attended a 1-day educational workshop designed to enhance knowledge and skills to engage in outdoor walking and prevent falls. After the workshop, participants were stratified by study site and participant type (enrolled as an individual or a dyad) and randomized into either the 10-week OWG program (n = 98) or the 10-week weekly reminders group (n = 92) [18]. Outcome evaluations were conducted at baseline, 3 months, 5.5 months, and 12 months. This study focused on the dose-response relationship between attendance in the OWG and improvement in health. Therefore, we limited our analytic sample to participants who were randomly assigned to the OWG program, remained in the study at the 3-month follow-up, and did not have missing data on all seven health outcomes at that time (n = 76). Health sciences research ethics boards at the University of Toronto, University of Manitoba, University of Alberta, and McGill University approved the trial protocol and participants provided written informed consent prior to baseline assessment. The research ethics board at the University of Toronto approved this analysis.

### Participants

Individuals were considered eligible for the trial if they were adults aged 65 years and older, living independently in the community, who self-reported difficulty walking outdoors but affirmed the capacity to walk at least one block (50m) with or without a walking aid, expressed willingness to sign a liability waiver or obtain physician clearance for exercise, exhibited mental competency (scoring at least 18 out of 22 on the Mini-Mental State Exam telephone version), were available to participate in the workshop and at least 5 weeks of the OWG program, and were able to speak and understand English. Individuals were excluded if they met the physical activity recommendation of 150 minutes per week, were receiving rehabilitation to improve walking, or at high risk for falls according to the American Geriatrics Society/British Geriatrics Society criteria [18]. We recruited by advertising with local newspapers, radio stations, senior’s centres, residences, and charities.

### Intervention: Outdoor walk group program

Based on a conceptual framework of community mobility [22], the OWG program was designed to enhance older adults’ competency in multiple dimensions of mobility such as walking distance, walking speed, postural transitions, and external physical load, within an outdoor environment [16]. The program involved attending two 1-hour sessions of group-based outdoor walking in large parks each week for a duration of 10 weeks (maximum of 20 sessions). The OWG program was progressive, task-specific, and implemented during summer months (June–August). Each session consisted of a 10-minute warm up, a distance walk, practice of a specific outdoor walking skill, a second distance walk, and a 10-minute cool down. Each OWG had a leader (physiotherapist or kinesiologist), who was authorized to adjust the program’s difficulty to ensure an optimal level of challenge for the participants [19]. Leaders were supported by additional trained staff to ensure a 1:3 ratio of OWG facilitators (leader/assistant) to participants. The 10-week duration of the OWG program is aligned with extant literature, which indicates that most group-based walking interventions for older adults range from 2 to 3 months [23]. After each OWG session, group leaders completed standardized forms to document implementation of session activities and participant attendance [19]. For the analysis of this study, participants were grouped into three categories based on attendance tertiles, which reflected the tri-modal distribution of attendance in the OWG program. The first, second, and third tertiles corresponded to participants attending 0–9, 10–15, and 16–20 total OWG sessions, respectively.

### Data collection

Information on individual characteristics was collected at baseline [16]. Participants self-reported their age (years), sex (male vs. female), highest level of education, and use of walking aids. The Charlson Comorbidity Index [24] was used to assess participants’ diagnosis of 18 comorbidities such as diabetes, hypertension, cancer, and heart diseases at baseline. The final weighted score ranges from 0 to 39, with higher scores indicating the presence of greater and more severe comorbidities.

### Performance-based measures

Walking endurance was assessed using the 6-minute walk test [25]. Participants received instructions to walk as far as possible in six minutes by walking back and forth along a straight, 30-meter walkway. Participants were asked to complete the test using their assistive devices and corrective eyewear as applicable. The maximum distance walked within six minutes was documented in meters. The 6-minute walk test demonstrates excellent test-retest reliability (intra-class correlation coefficient (ICC) = 0.95) and is considered a valid test of physical functioning and endurance among older adults [26,27].

Walking speed was assessed using the 10-meter walk test [28]. Participants were instructed to walk over a 14-meter walkway twice, once at a comfortable and once at a fast pace. Participants were asked to use their usual assistive devices and corrective eyewear to complete the test as applicable. The time taken to walk the central 10 meters was documented in seconds and used to calculate comfortable and fast walking speed (meter/second; m/s). The 10-meter walk test demonstrates excellent test-test reliability (ICCs = 0.96–0.98) and is recommended for clinical assessment of walking speed among older adults [29,30].

Balance was evaluated using the Mini Balance Evaluation System test (mini-BESTest) [31]. The mini-BESTest is a 14-item test developed to assess four balance domains [32]. Scores for anticipatory postural adjustments, reactive postural control, and sensory orientation range from 0 to 6, whereas the score for dynamic gait ranges from 0 to 10. Higher scores indicate greater balance in the respective subsystem. A total summed score, ranging from 0 to 28, is calculated to measure overall balance function. The mini-BESTest demonstrates good to excellent reliability (ICC > 0.90). Evidence of construct validity has been reported [31,33].

Lower extremity strength was measured using the 30-second sit-to-stand test [34]. Participants were instructed to sit in the middle of the chair, place arms folded across the chest, keep their feet placed on the floor, and repeat rising to a full standing position and sitting back down. The number of stands completed in 30 seconds was documented. The 30-second sit-to-stand test has shown excellent test-retest reliability (ICC = 0.95) and validity among community-dwelling older adults [34,35].

### Self-report questionnaires

Walking self-efficacy was evaluated using the ambulatory self-confidence questionnaire [36]. A total of 22 items are used to measure how confident participants are in their ability to walk in different environmental situations. Participants rated their responses to each item on a 10-point scale (0, not at all confident to 10, extremely confident). The total score is calculated as the mean of item scores and ranges from 0 to 10, with higher scores indicating greater confidence with walking ability. The ambulatory self-confidence questionnaire demonstrates excellent internal consistency (Cronbach’s α = 0.95) and test-retest reliability (ICC = 0.92) among community-dwelling older adults [36].

Emotional well-being was evaluated using the emotional well-being scale of the RAND 36-Item Health Survey (RAND-36) [37]. Participants answered five questions on their emotions and experiences over the past four weeks. Total scores range from 0 to 100, with higher scores indicating better emotional well-being. The RAND-36 Health Survey was adapted from the instruments administered in the Medical Outcome Study [37], and its emotional well-being scale has demonstrated internal consistency and reliability (Cronbach’s α = 0.90).

### Statistical analysis

Descriptive statistics were computed to describe participant characteristics and measures of physical and mental health at baseline in each attendance tertile groups (i.e., attended 0–9, 10–15, and 16–20 sessions). The tertile-based approach was adopted to account for the tri-modal distribution of OWG attendance observed in the GO-OUT trial. This approach enables clearer comparisons across low, moderate, and high levels of program engagement that could otherwise be obscured by using raw attendance data. We conducted nonparametric Kruskal-Wallis and chi-squared tests to compare differences in baseline characteristics and health outcome measures between attendance tertile groups. Wilcoxon signed-rank tests were used to examine changes in health outcomes from baseline to 3 months for each tertile group.

To examine potential forms of dose-response relationships between attendance and health improvement among older adults participating in the OWG program, we applied three established methodologies from the health sciences literature. These approaches, measuring the extent of improvement [38], the odds of improvement [39], and the overall count of health outcome improvements [40], have been used in similar studies focusing on older adults and other populations. *Extent of improvement*: First, we calculated an absolute change score on each measure of the seven health outcomes (walking endurance, comfortable and fast walking speed, balance, lower extremity strength, walking self-efficacy, and emotional well-being) from baseline to 3 months, where a positive change score indicates an improvement in the respective outcome. We employed linear regression models, using change scores as the dependent variable, and regressed them against the three attendance tertile groups. This allowed us to investigate whether higher attendance was associated with a greater extent of improvement in physical and mental health. A total of seven regression models were tested, with each focusing on a specific health outcome. We reported Hedges’ *g* effect sizes to provide insights into the magnitude of the dose-response relationship tested, with values of 0.15, 0.40, 0.75 representing small, medium, and large effects [41]. *Odds of improvement*: Second, we created a binary response variable to represent improvement (yes for change score > 0; no for change score ≤ 0) in each of the seven health outcomes. Separate logistic regression models were implemented to compare the odds of experiencing improvement in each health outcome across the three attendance groups. *Overall count of improvement in health*: Third, an overall count of improved health outcomes was created for each participant. A score of 0 suggests no improvement was found, while a score of 7 indicates improvement in all seven physical and mental health outcomes. A Poisson regression model was used to assess whether the number of improved health outcomes increased as a function of attendance in the OWG program. All models were adjusted for participant sex and study site, which were the two characteristics that differed across the attendance groups.

Recognizing that our analytic sample was restricted to 76 out of 98 OWG participants who remained in the study at 3 months and had data available for one or more of the seven health outcomes, we followed established procedure and employed the two-stage Heckman correction [42–44] to account for the impact of missing data and potential selection bias. Specifically, we first computed the inverse Mills ratio based on the full sample of older adults who were assigned to the OWG (n = 98) using a probit model. This ratio was then included in the regression models as an explanatory variable to adjust for non-selection hazards [42]. We conducted sensitivity analysis to compare findings with and without the Heckman correction. A two-tailed p-value less than 0.05 was considered statistically significant. All statistical analyses were performed using R software (Version 4.0.5).

## Results

### Descriptive statistics

Among the 98 older adults randomized to the OWG program, 78 remained in the study at the 3-month follow-up (79.6%). Among these, 2 participants had missing data on all seven health outcomes at the 3-month evaluation and were excluded from the analysis. Table 1 presents baseline participant characteristics and scores on measures of health outcomes for the entire sample and by level of outdoor walk group attendance of the 76 older adults included in the dose-response analysis. Mean±standard deviation age was 74.9 ± 6.6 years, and 72% of the participants were female. Prior to the OWG program, the mean distance that participants achieved on the 6-minute walk test was 363.2 ± 92.7 meters. The average walking speed was 1.09 ± 0.24 m/s at a comfortable pace and 1.43 ± 0.31 m/s at a fast pace. The mean score of mini-BESTest, 30-second sit-to-stand test, ambulatory self-confidence questionnaire, and RAND-36 emotional well-being scale, was 20.6 ± 4.7 (out of 28), 8.1 ± 3.5, 7.9 ± 1.6 (out of 10), and 77.1 ± 16.5 (out of 100), respectively. Upon closer examination of the three attendance tertile groups, participants from site 2 (56%) and site 4 (53%) attended 16 or more OWG sessions more frequently than those from site 1 (17%) and site 3 (17%) (*p* = 0.017). Fifty-seven percent of the 21 male participants but only 29% of the 55 female participants attended 16 or more OWG sessions (*p* = 0.006). For physical performance, participants who attended 0–9 OWG sessions showed a trend toward greater baseline walking endurance (*p* = 0.067) and had significantly faster baseline walking speed (*p* = 0.030) compared to those attending a greater number of sessions. Remaining baseline individual and clinical characteristics did not differ across the three attendance tertile groups. S1 Table shows that the baseline characteristics of OWG participants who remained in the GO-OUT study at 3 months (n = 78) compared to those who withdrew (n = 20) were similar with one exception: compared to participants who withdrew, individuals remaining in the study reported higher average emotional well-being at baseline (*p* = 0.032).

**Table 1:** Participant characteristics and scores on health outcome measures at baseline by level of outdoor walk group attendance.

Participant characteristic (units or scoring)	Pooled (n = 76)	OWG attendance tertile*			
		1^st^ tertile (n = 15)	2^nd^ tertile (n = 33)	3^rd^ tertile (n = 28)	*p* value
			Mean ± SD/ n (%)		
Study site					.017
Site 1	18 (24)	7 (39)	8 (44)	3 (17)	
Site 2	25 (33)	2 (8)	9 (36)	14 (56)	
Site 3	18 (24)	5 (28)	10 (56)	3 (17)	
Site 4	15 (20)	1 (7)	6 (40)	8 (53)	
Participant type					.307
Individual	60 (79)	14 (23)	24 (40)	22 (37)	
Dyad	16 (21)	1 (6)	9 (56)	6 (38)	
Cohort					.634
2018–19	29 (38)	7 (24)	13 (45)	9 (31)	
2019–20	47 (62)	8 (17)	20 (43)	19 (40)	
Age (years)	74.9 ± 6.6	74.3 ± 6.5	74.5 ± 6.4	75.8 ± 7.1	.806
Sex					.006
Male	21 (28)	0 (0)	9 (43)	12 (57)	
Female	55 (72)	15 (27)	24 (44)	16 (29)	
Education attainment					.414
Secondary school or lower	19 (25)	3 (16)	6 (32)	10 (53)	
Some college	27 (36)	4 (15)	13 (48)	10 (37)	
Bachelor’s degree or higher	30 (39)	8 (27)	14 (47)	8 (27)	
Uses a walking aid	20 (26)	3 (15)	11 (55)	6 (30)	.550
Charlson comorbidity index (0–39)	2.1 ± 2.0	2.1 ± 3.1	2.2 ± 1.6	1.9 ± 1.7	.433
6-minute walk test (m)	363.2 ± 92.7	413.0 ± 83.9	344.5 ± 92.7	360.1 ± 90.8	.067
10-meter walk test at comfortable pace (m/s)	1.09 ± 0.24	1.25 ± 0.23	1.06 ± 0.22	1.04 ± 0.24	.030
10-meter walk test at fast pace (m/s)	1.43 ± 0.31	1.59 ± 0.30	1.38 ± 0.28	1.41 ± 0.34	.086
Mini-BESTest (0–28)	20.6 ± 4.7	21.4 ± 4.3	20.2 ± 4.7	20.6 ± 5.0	.689
30-second sit-to-stand (# stands)	8.1 ± 3.5	8.1 ± 3.5	8.1 ± 3.6	8.1 ± 3.6	.997
ASCQ (0–10)	7.9 ± 1.6	8.1 ± 1.6	7.6 ± 1.8	8.2 ± 1.4	.386
RAND-36 emotional well-being (0–100)	77.1 ± 16.5	71.5 ± 20.2	80.1 ± 13.5	76.6 ± 17.3	.380

*Note*: OWG = outdoor walk group; Mini-BESTest = Mini Balance Evaluation System test; ASCQ = ambulatory self-confidence questionnaire; SD = standard deviation; m = meters; m/s = meters/second.

* The 1^st^ tertile group attended 0–9 sessions; the 2^nd^ tertile group attended 10–15 sessions; the 3^rd^ tertile group attended 16–20 sessions. Tertiles were calculated based on all enrolled participants in the outdoor walk group program (n = 98). Values in the parenthesis indicate row percentages.

### Mean change in scores on physical and mental health outcome measures by outdoor walk group attendance

Table 2 presents the scores at baseline and 3 months, and change in score from baseline to 3 months, on seven physical and mental health outcome measures, stratified by OWG attendance tertile groups. After the 10-week OWG program, participants in all three attendance groups demonstrated significant improvement in 6-minute walk test performance, with improvements ranging from 15.7 meters to 37.3 meters. The second (10–15 sessions) and third (16–20 sessions) tertile groups also showed significant improvements in the 10-meter walk test at a comfortable and fast pace. On the 30-second sit-to-stand test, participants in the first (0–9 sessions) and third (16–20 sessions) tertile groups performed approximately one additional stand from baseline to 3 months. No significant changes were observed on measures of walking self-efficacy, balance or emotional well-being in any of the OWG attendance tertile groups.

**Table 2:** Change in score on seven health outcome measures from baseline to 3 months by level of outdoor walk group attendance.

Health outcome measure (units or scoring)	Scores on health outcome measures			
	0 Month (A)	3 Months (B)	Change (B-A)	*p* value*
	1^st^ attendance tertile group (0–9 sessions)			
	Mean ± SD			
6-minute walk test (m)	413.0 ± 83.9	437.1 ± 93.7	15.7 ± 71.2	.025
10-meter walk test at comfortable pace (m/s)	1.25 ± 0.23	1.25 ± 0.26	0.01 ± 0.25	.244
10-meter walk test at fast pace (m/s)	1.59 ± 0.30	1.58 ± 0.36	–0.01 ± 0.21	1.000
Mini-BESTest (0–28)	21.4 ± 4.3	23.1 ± 4.2	1.7 ± 3.4	.075
30-second sit-to-stand (# stands)	8.1 ± 3.5	9.3 ± 3.2	1.3 ± 2.2	.048
ASCQ (0–10)	8.1 ± 1.6	8.4 ± 1.4	0.3 ± 1.3	.330
RAND-36 emotional well-being (0–100)	71.5 ± 20.2	72.5 ± 19.5	1.1 ± 15.6	1.000
	**2**^nd^ **attendance tertile group (10–15 sessions)**			
6-minute walk test (m)	344.5 ± 92.7	369.1 ± 88.7	25.8 ± 49.0	<.001
10-meter walk test at comfortable pace (m/s)	1.06 ± 0.22	1.16 ± 0.18	0.09 ± 0.18	.012
10-meter walk test at fast pace (m/s)	1.38 ± 0.28	1.48 ± 0.27	0.10 ± 0.23	.016
Mini-BESTest (0–28)	20.2 ± 4.7	19.8 ± 5.5	–0.4 ± 3.8	.780
30-second sit-to-stand (# stands)	8.1 ± 3.6	8.9 ± 5.0	0.8 ± 2.8	.085
ASCQ (0–10)	7.6 ± 1.8	7.9 ± 1.6	0.4 ± 1.4	.058
RAND-36 emotional well-being (0–100)	80.1 ± 13.5	79.3 ± 14.5	–0.9 ± 14.7	.781
	**3**^rd^ **attendance tertile group (16–20 sessions)**			
6-minute walk test (m)	360.1 ± 90.8	399.3 ± 103.8	37.3 ± 49.5	<.001
10-meter walk test at comfortable pace (m/s)	1.04 ± 0.24	1.17 ± 0.22	0.12 ± 0.17	.001
10-meter walk test at fast pace (m/s)	1.41 ± 0.34	1.53 ± 0.33	0.13 ± 0.16	<.001
Mini-BESTest (0–28)	20.6 ± 5.0	20.7 ± 5.7	0.1 ± 3.2	.785
30-second sit-to-stand (# stands)	8.1 ± 3.6	9.6 ± 4.2	1.5 ± 1.8	.001
ASCQ (0–10)	8.2 ± 1.4	8.5 ± 1.2	0.4 ± 1.3	.241
RAND-36 emotional well-being (0–100)	76.6 ± 17.3	80.3 ± 12.7	3.7 ± 13.8	.460

*Note*: Mean ± standard deviation (SD) are reported for each health outcome measure and for each attendance tertile group at baseline and 3 months. Change scores were calculated by subtracting baseline scores from the 3-month scores.

*Wilcoxon signed-rank tests were used to examine whether mean changes in health outcome measures were statistically significant from baseline to 3 months.

### Influence of outdoor walk group attendance on the extent of improvement in health

The associations between attendance in the OWG program and extent of improvement in the seven physical and mental health outcomes are summarized in Table 3. Compared to those who attended 0–9 OWG sessions (1^st^ tertile), participants who attended 16–20 sessions (3^rd^ tertile) exhibited, on average, a 56.33-meter greater improvement in the 6-minute walk test performance from baseline to 3 months that was statistically significant (95% CI: 17.29, 95.36, *p* = 0.005). Participants attending 16–20 sessions achieved on average, a 28.28-meter greater improvement in the 6-minute walk test performance from baseline to 3 months than those who attended 10–15 sessions, but this difference was not statistically significant (*b* = 28.28, 95% CI: −0.26, 56.82, *p* = 0.052). The effect sizes of the dose-response relationships observed were small in magnitude (Hedges’ *g* range: 0.23–0.36; see S2 Table).

**Table 3:** Associations between outdoor walk group attendance and the extent of improvement in health outcome measures from baseline to 3 months.

Health outcome measures (units or scoring)	Comparisons between OWG attendance tertiles		
	2^nd^ tertile (10–15 sessions) vs 1^st^ tertile (0–9 sessions)	3^rd^ tertile (16–20 sessions) vs 1^st^ tertile (0–9 sessions)	3^rd^ tertile (16–20 sessions) vs 2^nd^ tertile (10–15 sessions)
	Unstandardized regression coefficient *b* [95% confidence interval]		
6-minute walk test (m)	28.05 [−6.28, 62.38]	56.33 [17.29, 95.36] **	28.28 [−0.26, 56.82] ^†^
10-meter walk test at comfortable pace (m/s)	0.10 [−0.03, 0.22]	0.15 [0.01, 0.29] *	0.06 [−0.04, 0.16]
10-meter walk test at fast pace (m/s)	0.14 [0.01, 0.28] *	0.18 [0.03, 0.34] *	0.04 [−0.07, 0.15]
Mini-BESTest (0–28)	−1.26 [−3.57, 1.05]	−0.09 [−2.76, 2.56]	1.17 [−0.74, 3.08]
30-second sit-to-stand (# stands)	−0.42 [−1.97, 1.13]	0.17 [−1.62, 1.96]	0.59 [−0.69, 1.88]
ASCQ (0–10)	0.38 [−0.48, 1.23]	0.50 [−0.50, 1.50]	0.13 [−0.60, 0.85]
RAND-36 emotional well-being (0–100)	−4.08 [−13.86, 5.70]	−0.50 [−11.87, 10.87]	3.58 [−4.60, 11.76]

*Note*: The regression coefficients are in reference to the lower tertile/attendance group, with positive values suggesting potential dose-response relationships. All regression models were adjusted for participant sex, study site, and the Heckman correction.

†*p* < 0.10;

* *p* < 0.05;

**p < 0.01.

**Fig 1:**
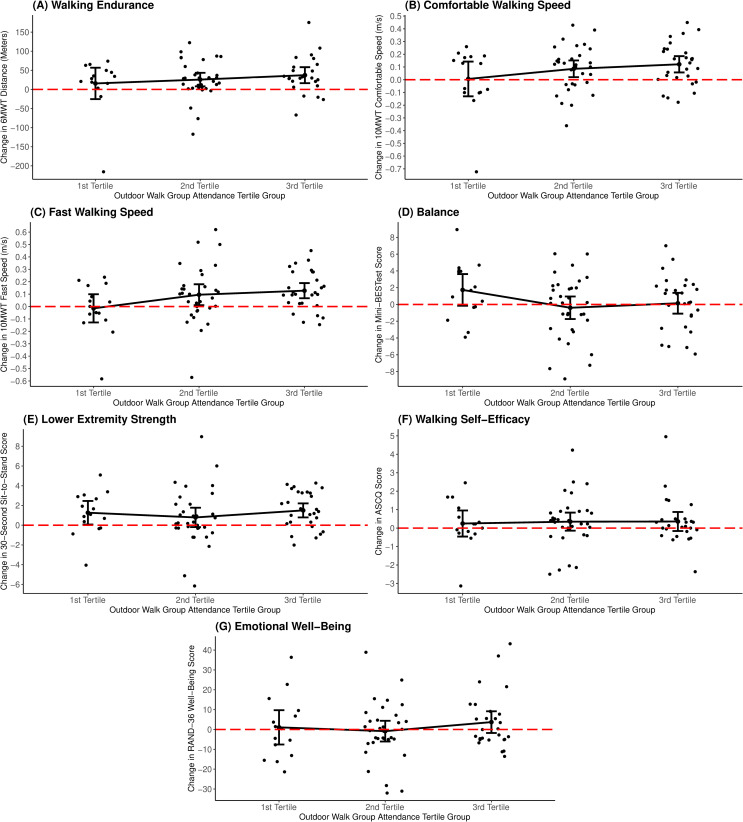
Changes in measures of physical and mental health from baseline to 3 months across three outdoor walk group attendance groups. *Note*: The 1^st^ tertile group attended 0–9 sessions; the 2^nd^ tertile group attended 10–15 sessions; the 3^rd^ tertile group attended 16–20 sessions. Dots above the horizontal dashed line indicate an improvement (change score > 0) in the respective health outcome measure from baseline to 3 months.

Participants attending 10–15 OWG sessions exhibited a significantly greater extent of improvement in fast walking speed compared to those with 0–9 sessions (*b* = 0.14, 95% CI: 0.01, 0.28, *p* = 0.040). Similarly, those attending 16–20 sessions demonstrated significantly greater improvements in both comfortable walking speed (*b* = 0.15, 95% CI: 0.01, 0.29, *p* = 0.034) and fast walking speed (*b* = 0.18, 95% CI: 0.03, 0.34, *p* = 0.020) compared to participants with 0–9 sessions. The effects observed were medium to strong in magnitude (Hedges’ *g* range: 0.49–0.80). There were no significant relationships found between attendance and absolute changes in measures of balance, lower extremity strength, walking self-efficacy, and emotional well-being. Fig 1 presents results without covariate adjustment.

### Influence of outdoor walk group program attendance on the odds of improvement in health

Table 4 presents the associations between OWG attendance and the odds of experiencing an improvement in health. Results from the adjusted logistic regression models showed that compared to those attending only 0–9 OWG sessions, the odds of experiencing an improvement in comfortable walking speed from baseline to 3 months tended to be about 7 times larger among participants who attended 16–20 OWG sessions (Odds ratio [OR] = 7.06, 95% CI: 1.13, 57.83, *p* = 0.047). The odds of experiencing an improvement in fast walking speed were 10 times greater among participants who attended 16–20 OWG sessions than those attending 0–9 sessions (OR = 10.06, 95% CI: 1.75, 72.00, *p* = 0.014) and 3 times larger than those attending 10–15 sessions but this comparison was not statistically significant (OR = 3.19, 95% CI: 0.82, 12.50, *p* = 0.095). Likewise, participants attending 10–15 OWG sessions tended to have higher odds of improvement in walking self-efficacy, as measured by the ambulatory self-confidence questionnaire, compared to those attending 0–9 sessions but this comparison was not statistically significant (OR = 3.88, 95% CI: 0.94, 17.43, *p* = 0.066). The unadjusted percentages of participants experiencing an improvement in health across the three attendance tertile groups are presented in S1 Fig.

**Table 4:** Associations between outdoor walk group attendance and the odds of experiencing improvement in health outcome measures from baseline to 3 months.

	Comparisons between OWG attendance tertiles		
Health outcome measure	2^nd^ tertile (10–15 sessions) vs 1^st^ tertile (0–9 sessions)	3^rd^ tertile (16–20 sessions) vs 1^st^ tertile (0–9 sessions)	3^rd^ tertile (16–20 sessions) vs 2^nd^ tertile (10–15 sessions)
	Odds ratio [95% confidence interval]		
6-minute walk test (m)	2.36 [0.22, 23.70]	5.42 [0.45, 73.23]	2.29 [0.39, 13.48]
10-meter walk test at comfortable pace (m/s)	3.49 [0.70, 20.94]	7.06 [1.13, 57.83] *	2.02 [0.55, 7.47]
10-meter walk test at fast pace (m/s)	3.15 [0.76, 14.28]	10.06 [1.75, 72.00] *	3.19 [0.82, 12.50] ^†^
Mini-BESTest (0–28)	0.66 [0.16, 2.66]	0.96 [0.20, 4.78]	1.45 [0.47, 4.48]
30-second sit-to-stand (# stands)	0.36 [0.08, 1.47]	0.83 [0.14, 4.54]	2.30 [0.72, 7.39]
ASCQ (0–10)	3.88 [0.94, 17.43] ^†^	3.06 [0.61, 17.15]	0.79 [0.24, 2.65]
RAND-36 emotional well-being (0–100)	0.61 [0.15, 2.45]	0.76 [0.15, 3.79]	1.25 [0.40, 3.95]

*Note*: The odds ratios are in reference to the lower tertile/attendance group, with values greater than 1 suggesting potential dose-response relationships. All regression models were adjusted for participant sex, study site, and the Heckman correction.

†*p* < 0.10;

* *p* < 0.05.

### Influence of outdoor walk group program attendance on the number of health outcomes improved

Table 5 presents results from the Poisson regression model of the relationship between OWG program attendance and the overall count of improved health outcomes from baseline to 3 months. The total number of improvements on the measures of seven health outcomes increased with the number of OWG sessions attended: 4.00 ± 1.46 for 0–9 sessions, 4.03 ± 1.69 for 10–15 sessions, and 4.43 ± 1.50 for 16–20 sessions; the differences across tertile groups, however, were not statistically significant. Analyses conducted with and without the Heckman correction yielded similar results. The findings from the models without the correction are presented in S3–S5 Tables.

**Table 5:** Associations between outdoor walk group attendance and the total count of improvement on health outcome measures from baseline to 3 months.

Outdoor walk group attendance	Total count of improvement on health outcome measures (Range: 0–7)	
	Mean ± SD	Incident rate ratio [95% CI]
1^st^ tertile (0–9 sessions)	4.00 ± 1.46	1.00 (Reference)
2^nd^ tertile (10–15 sessions)	4.03 ± 1.69	1.09 [0.79, 1.52]
3^rd^ tertile (16–20 sessions)	4.43 ± 1.50	1.26 [0.87, 1.84]
	Comparisons between 2^nd^ and 3^rd^ tertiles	
2^nd^ tertile (10–15 sessions)		1.00 (Reference)
3^rd^ tertile (16–20 sessions)		1.16 [0.89, 1.50]

*Note*: CIs = confidence interval. The incident rate ratios are in reference to the lower tertile/attendance group, with values greater than 1 suggesting potential dose-response relationships. The Poisson regression model was adjusted for participant sex, study site, and the Heckman correction.

## Discussion

This study investigated potential dose-response relationships between attendance in an OWG program and improvement on measures of seven physical and mental health outcomes among community-dwelling older adults with mobility limitations. Results showed that, compared to participants with lower attendance levels, those with higher attendance exhibited greater improvement in walking endurance, comfortable walking speed, and fast walking speed, from baseline to post-intervention. Moreover, participants with higher attendance had an increased odds of experiencing improvement in comfortable and fast walking speed.

Overall, our findings align with the existing body of literature documenting the health benefits of walking interventions [4,23,45–47]. For example, a recent systematic review and meta-analysis of nine randomized controlled trials reported that walking-only interventions significantly improved walking endurance and health-related quality of life among community-dwelling older adults [47]. Our study extends this body of work by focusing on a park-based, supervised, task-oriented and progressive OWG program and exploring the role of session attendance in driving improvements in older adults’ health. We found that participants who attended 16–20 park-based walking sessions demonstrated a 56.3-meter greater improvement in walking endurance, as measured by the 6-minute walk test, compared to those who attended only 0–9 sessions. Our findings are consistent with prior work reported by Dondzila et al. [48], showing a gradual improvement in 6-minute walk test performance as older adults increased their walking activity from 2,500 to 7,500 steps per day. Notably, participants in our study who were in the high attendance tertile (16–20 sessions) showed a mean improvement of 37.3 meters in 6-minute walk test performance from baseline to 3 months, which exceeds the minimal clinically important difference (MCID) of 30.5 meters [49]. In contrast, participants in the lower attendance tertiles (0–15 sessions) did not achieve improvements that surpassed the MCID. Our study presents preliminary evidence suggesting that participating in 16 or more sessions of a park-based OWG program over a period of 10 weeks may be needed to provide sufficient training for achieving improvements in walking endurance among older adults with mobility limitations.

Our study is among the first to identify dose-response relationships between attendance in OWG sessions and improvements in both comfortable and fast walking speed within a community setting. Although evidence directly examining this relationship remains limited, previous research has explored related concepts [50–53]. For instance, a recent randomized controlled trial led by Klassen and colleagues [51] showed that acute stroke patients receiving high-dose exercise interventions (40 sessions) in an indoor hospital setting exhibited significant improvements in walking speed compared to those receiving standard stroke care, while no such improvements were observed among those with low-dose interventions (20 sessions). Similarly, a meta-analysis involving 2,054 community-dwelling older adults suggested that high-dose therapeutic exercise interventions, defined as three 60-minute sessions per week, had a significant positive impact on improving walking speed, whereas low-dose interventions did not yield similar effects [52]. It is worth noting that participants in our study who attended 16–20 OWG sessions demonstrated an average improvement of 0.12 ± 0.17 m/s in comfortable walking speed and 0.13 ± 0.16 m/s in fast walking speed from baseline to 3 months. The mean improvement in comfortable walking speed surpasses the minimum MCID value of 0.10 m/s identified in prior studies involving diverse patient populations, including older adults with stroke, hip fracture, and multiple sclerosis [54]. Moreover, the extent of change in fast walking speed among participants attending 16–20 OWG sessions also exceeds the minimum MCID value of 0.12 m/s reported in a recent systematic review involving community-dwelling older adults [55]. These findings underscore that greater participation in an outdoor walk group program is associated with not only statistically significant, but also clinically meaningful, improvements in walking capacities.

To delve deeper into the impact of attendance in the OWG program, our study expanded its scope beyond merely measuring changes in health. We incorporated two additional approaches to operationalize dose-response and investigate how attendance would affect both the odds and overall count of improvements in health. Through this approach, we identified emerging evidence suggesting that, although not statistically significant, attending a greater number of OWG sessions may positively influence older adults’ walking self-efficacy. As a range of methodological approaches have been developed to examine dose-response relationships across fields of health sciences [38–40], further research utilizing distinct operational definitions of response is warranted to evaluate the potential benefits of outdoor walking interventions on health of older adults.

Contrary to expectations, our analysis revealed no significant dose-response relationship between attendance in the OWG program and improvement in balance, lower extremity strength, and emotional well-being. Several factors may shed light on these findings. First and foremost, the OWG program focused primarily on task-specific training designed to enhance older adults’ competency and skills in outdoor ambulation, including walking, turning, and stepping sideways [16]. Task-specificity of training may help to explain why we observed associations between attendance and improvements in walking capacity, but not in other general health outcomes. Given the focus of our intervention on walking distance, with two distance walks completed each session [19], it is not surprising that associations between attendance and improvements in walking endurance and speed were observed. Second, a meta-analysis including 1,252 participants across 10 studies demonstrated a U-shaped relationship between nature-based exercise and mental health [56]. They found that the largest benefits of nature and green exercise on mental health, measured by self-esteem and total mood disturbance, were observed at the shortest duration of 5 minutes per day. These benefits diminished as the duration increased to 10–60 min and half-day sessions but rose again for the whole day duration. This phenomenon may partly explain the lack of clear dose-response relationship between OWG attendance and emotional well-being in our study. Interestingly, an experimental study conducted by Li and colleagues showed contrasting effects of outdoor walking based on timing and location [57]. They found that while walking in an urban environment during nighttime was associated with positive effects on blood pressure, emotions and moods among middle-aged and older adults, walking in the same environment during daytime had negative effects on health potentially due to urban stressors such as noise, crowding, and air pollution. As our OWG program was administered in parks within four large Canadian cities during the day, it is possible that the timing and location of the walks, and the need to obtain transportation to parks, may have influenced the observed outcomes. Taken together, these insights highlight the complexity of designing and implementing outdoor walking interventions for older adults, suggesting the need to leverage an integrated paradigm that accounts for the diverse individual, environmental, and contextual factors involved in these initiatives [20].

This study possesses numerous strengths. Our OWG program is among the first that incorporates task-specific training of outdoor activities targeted towards enhancing community mobility skills among older adults [8]. In addition, our study spanned four major cities across Canada, which strengthens the external validity of our findings and allows generalization to similar urban environments. Furthermore, the inclusion of a broad range of outcome measures and operational definitions of response contributes to a better understanding of the impact of outdoor walking interventions on older adults’ health. However, findings should be interpreted within the context of their limitations. For example, although we observed gradients in the association between OWG attendance and total count of improved health outcomes, the lack of significance may result from limited sample size compared to prior studies involving outdoor community ambulation interventions among older adults [8]. Importantly, our study targeted older adults with mobility limitations. Even though participants self-reported difficulty walking outdoors, their baseline comfortable (1.09 ± 0.24 m/s) and fast walking speed (1.43 ± 0.31 m/s) were mostly above the average requirements for community ambulation (0.44 to 1.32m/s) [58]. This discrepancy suggests that our sample may have included individuals who possessed relatively higher functional capacity, potentially limiting the generalizability of findings to older adults with more severe mobility impairments. We also noted that attendance levels varied widely across the four study sites. Our planned process evaluation documented several reasons for non-attendance, including illness, vacation, transportation difficulties, and other commitments [18,19]. To improve participation, attention should be given to developing strategies that address both individual and structural barriers and promote consistent engagement among older adults across diverse urban settings [11–13,59]. Collectively, our study offers practical implications for the design, implementation, and promotion of community-based exercise interventions aimed at supporting the health of older adults [60,61]. From a design perspective, our findings underscore the necessity of tailoring programs to meet the needs, abilities, and preferences of older adults to maximize their engagement. From a promotional standpoint, healthcare providers and program developers may leverage the insights from this study to emphasize the importance of consistent attendance as a key factor in achieving meaningful improvements in walking capacity. Building on these findings, future research could explore the impact of attendance in different settings, such as long-term care facilities or through hybrid models that combine outdoor walking with online support. In parallel, investigating how attendance interacts with other dose parameters, such as walking intensity, frequency, and duration, represents another critical avenue for future research.

In conclusion, this study highlights the positive impact of attendance in a park-based, supervised, task-oriented and progressive OWG program on improving walking endurance, comfortable walking speed, and fast walking speed among older adults with mobility limitations. Our findings reinforce the importance of continued clinical and research endeavors to support participation and engagement in outdoor walking programs. Ultimately, these efforts will allow us to translate volume into value, enhancing the health and well-being for older adults.

## Supporting information

S1 Fig Raw percentage of participants who experienced improvement in physical and mental health outcomes from baseline to 3 months across three outdoor walk group tertile attendance groups. (PDF)

S1 Table Comparison of baseline characteristics between outdoor walk group participants who remained in the GO-OUT study and those who had withdrawn at 3 months. (PDF)

S2 Table Size of the effect of outdoor walk group attendance on the extent of improvement on health outcome measures from baseline to 3 months. (PDF)

S3 Table Associations between outdoor walk group attendance and the extent of improvement on health outcome measures from baseline to 3 months (without the Heckman correction). (PDF)

S4 Table Associations between outdoor walk group attendance and the odds of experiencing improvement on health outcome measures from baseline to 3 months (without the Heckman correction). (PDF)

S5 Table Associations between outdoor walk group attendance and total count of health outcomes with improved scores from baseline to 3 months (without the Heckman correction). (PDF)
