# Supplementary material for: Examining dose-response of an outdoor walk group program in the Getting Older Adults Outdoors (GO-OUT) trial
Source: PLoS One. 2025 Mar 13;20(3):e0309933. doi: 10.1371/journal.pone.0309933 (PMC11906069; doi:10.1371/journal.pone.0309933)
Supplement: S1 Fig — (PDF) [file pone.0309933.s001.pdf]

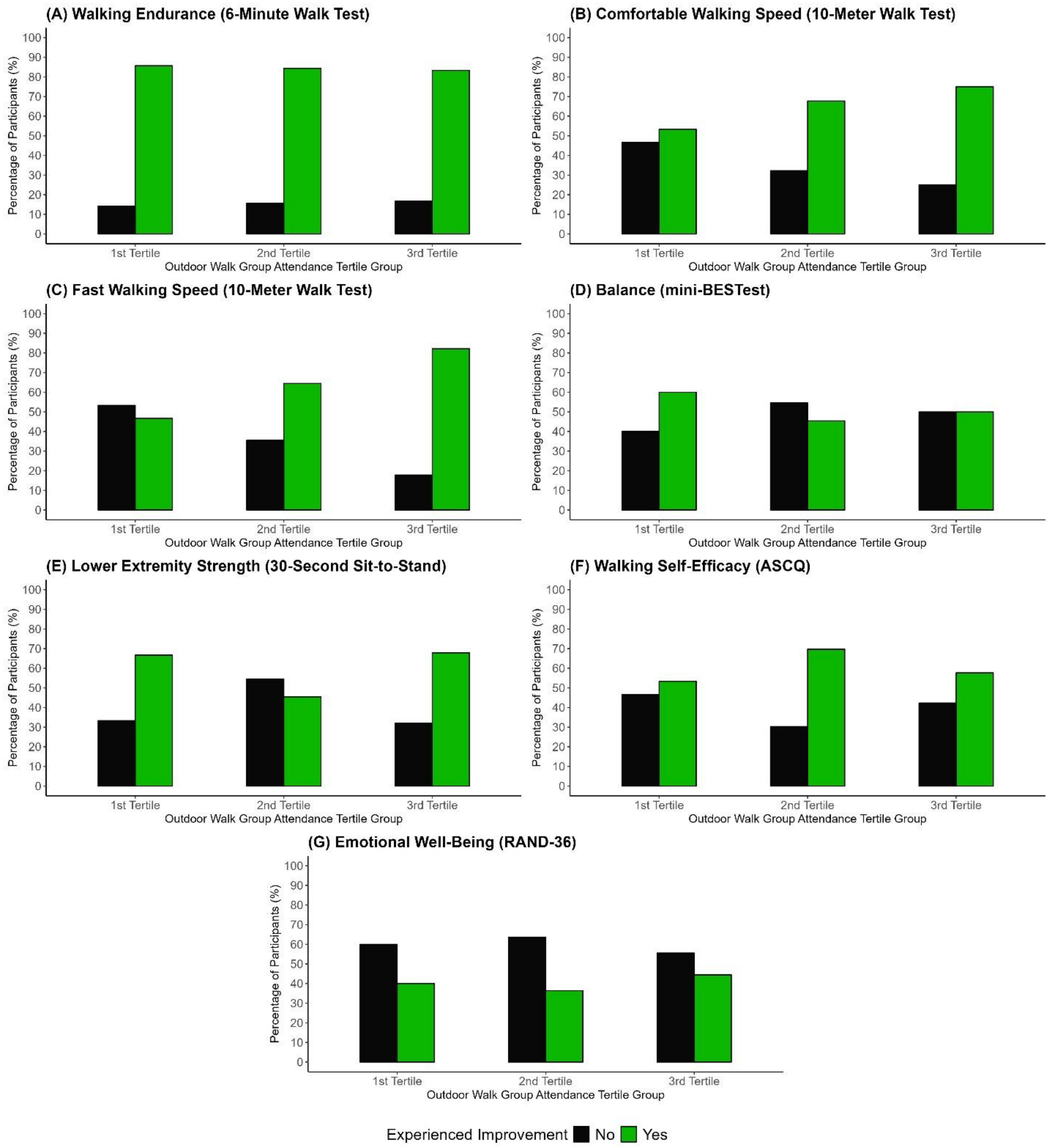

S1 Fig. Raw percentage of participants who experienced improvement in physical and mental health outcomes from baseline to 3 months across three outdoor walk group tertile attendance groups.

Note: The 1st tertile group attended 0–9 sessions; the 2nd tertile group attended 10–15 sessions; the 3rd tertile group attended 16–20 sessions.
