# Supplementary material for: Examining dose-response of an outdoor walk group program in the Getting Older Adults Outdoors (GO-OUT) trial
Source: PLoS One. 2025 Mar 13;20(3):e0309933. doi: 10.1371/journal.pone.0309933 (PMC11906069; doi:10.1371/journal.pone.0309933)
Supplement: S1 Table — (PDF) [file pone.0309933.s002.pdf]

**S1 Table**. Comparison of baseline characteristics between outdoor walk group participants who remained in the GO-OUT study and those who had withdrawn at 3 months

| Participant characteristic (units or scoring) | Participants remained in the GO-OUT Study (n = 78) | Withdrawals (n = 20) | *p* value |
|---|---|---|---|
| | Mean ± SD / n (%) | | |
| Study site | | | .364 |
| Edmonton | 19 (73) | 7 (27) | |
| Winnipeg | 15 (83) | 3 (17) | |
| Toronto | 19 (73) | 7 (27) | |
| Montreal | 25 (89) | 3 (11) | |
| Participant type | | | .757 |
| Individual | 61 (78) | 17 (22) | |
| Dyad | 17 (85) | 3 (15) | |
| Cohort | | | .063 |
| 2018-19 | 30 (91) | 3 (9) | |
| 2019-20 | 48 (74) | 17 (26) | |
| Age (years) | 74.8 ± 6.6 | 74.6 ± 8.2 | .889 |
| Sex | | | .144 |
| Male | 22 (92) | 2 (8) | |
| Female | 56 (76) | 18 (24) | |
| Educational attainment | | | .217 |
| Secondary or lower | 19 (90) | 2 (10) | |
| Some or completed college | 28 (82) | 6 (18) | |
| Bachelor’s degree or higher | 31 (72) | 12 (28) | |
| Uses a walking aid | 20 (71) | 8 (29) | .322 |
| Charlson comorbidity index (0–39) | 2.0 ± 2.0 | 2.1 ± 2.2 | .897 |
| 6-minute walk test (m) | 362.5 ± 92.8 | 345.2 ± 69.6 | .449 |
| 10-meter walk test at comfortable pace (m/s) | 1.09 ± 0.24 | 1.02 ± 0.25 | .198 |
| 10-meter walk test at fast pace (m/s) | 1.44 ± 0.31 | 1.31 ± 0.30 | .120 |
| Mini-BESTest (0–28) | 20.7 ± 4.7 | 18.6 ± 5.8 | .090 |
| 30-second sit-to-stand (# stands) | 8.2 ± 3.5 | 7.4 ± 4.3 | .380 |
| ASCQ (0–10) | 7.9 ± 1.6 | 7.2 ± 1.9 | .078 |
| RAND-36 emotional well-being (0–100) | 77.2 ± 16.4 | 68.0 ± 17.2 | .032 |

*Note*: Mini-BESTest = Mini Balance Evaluation System test, with higher scores indicating better overall balance function; ASCQ = ambulatory self-confidence questionnaire, with higher scores indicating greater confidence with walking ability; SD = standard deviation; m = meters; m/s = meters/second. Values in the parenthesis indicate row percentages.
