# Supplementary material for: Examining dose-response of an outdoor walk group program in the Getting Older Adults Outdoors (GO-OUT) trial
Source: PLoS One. 2025 Mar 13;20(3):e0309933. doi: 10.1371/journal.pone.0309933 (PMC11906069; doi:10.1371/journal.pone.0309933)
Supplement: S2 Table — (PDF) [file pone.0309933.s003.pdf]

S2 Table. Size of the effect of outdoor walk group attendance on the extent of improvement on health outcome measures from baseline to 3 months.

	Comparisons between OWG attendance tertile groups		
	2nd tertile (10–15 sessions) vs 1st tertile (0–9 sessions)	3rd tertile (16–20 sessions) vs 1st tertile (0–9 sessions)	3rd tertile (16–20 sessions) vs 2nd tertile (10–15 sessions)
Measures	Effect size Hedges' *g* [95% CI]		
6-minute walk test	0.18 [–0.46, 0.81]	0.36 [–0.31, 1.04]	0.23 [–0.30, 0.77]
10-meter walk test at comfortable pace	0.39 [–0.24, 1.01]	0.57 [–0.07, 1.22]	0.20 [–0.31, 0.72]
10-meter walk test at fast pace	0.49 [–0.15, 1.12]	0.80 [0.14, 1.45]	0.16 [–0.35, 0.68]
Mini-BESTest	–0.57 [–1.20, 0.06]	–0.48 [–1.12, 0.17]	0.15 [–0.36, 0.66]
30-second sit-to-stand	–0.18 [–0.80, 0.44]	0.12 [–0.52, 0.75]	0.29 [–0.22, 0.80]
ASCQ	0.07 [–0.54, 0.69]	0.08 [–0.56, 0.73]	0.01 [–0.51, 0.52]
RAND-36 emotional well-being	–0.13 [–0.74, 0.49]	0.18 [–0.46, 0.82]	0.31 [–0.20, 0.83]

Note: OWG = outdoor walk group; Mini-BESTest = Mini Balance Evaluation System test; ASCQ = Ambulatory Self-Confidence Questionnaire. The effect size can be interpreted as small (Hedges' *g* = 0.15), medium (*g* = 0.40), and large (*g* = 0.75).
