# Supplementary material for: Examining dose-response of an outdoor walk group program in the Getting Older Adults Outdoors (GO-OUT) trial
Source: PLoS One. 2025 Mar 13;20(3):e0309933. doi: 10.1371/journal.pone.0309933 (PMC11906069; doi:10.1371/journal.pone.0309933)
Supplement: S3 Table — (PDF) [file pone.0309933.s004.pdf]

S3 Table. Associations between outdoor walk group attendance and the extent of improvement on health outcome measures from baseline to 3 months (without the Heckman correction)

	Comparisons between OWG attendance tertile groups		
	2nd tertile (10–15 sessions)<br>vs<br>1st tertile (0–9 sessions)	3rd tertile (16–20 sessions)<br>vs<br>1st tertile (0–9 sessions)	3rd tertile (16–20 sessions)<br>vs<br>2nd tertile (10–15 sessions)
Measures	Unstandardized regression coefficient *b* [95% CIs]		
6-minute walk test	26.27 [–9.41, 61.95]	50.17 [9.89, 90.45] *	23.90 [–5.56, 53.36]
10-meter walk test at comfortable pace	0.08 [–0.05, 0.20]	0.12 [–0.02, 0.27] †	0.05 [–0.06, 0.15]
10-meter walk test at fast pace	0.13 [–0.01, 0.26] †	0.16 [0.01, 0.32] *	0.03 [–0.08, 0.14]
Mini-BESTest	–1.41 [–3.71, 0.90]	–0.32 [–2.97, 2.32]	1.08 [–0.83, 2.99]
30-second sit-to-stand	–0.35 [–1.89, 1.19]	0.29 [–1.48, 2.05]	0.64 [–0.64, 1.91]
ASCQ	0.31 [–0.55, 1.17]	0.39 [–0.61, 1.39]	0.08 [–0.65, 0.81]
RAND-36 emotional well-being	–3.79 [–13.46, 5.88]	–0.01 [–11.17, 11.15]	3.78 [–4.32, 11.88]

Note: CI = confidence intervals. The regression coefficients are in reference to the lower tertile/attendance group, with positive values suggesting potential dose-response relationships. All regression models were adjusted for participants' sex and study site. †p<.10; *p<.05
