# Supplementary material for: Examining dose-response of an outdoor walk group program in the Getting Older Adults Outdoors (GO-OUT) trial
Source: PLoS One. 2025 Mar 13;20(3):e0309933. doi: 10.1371/journal.pone.0309933 (PMC11906069; doi:10.1371/journal.pone.0309933)
Supplement: S4 Table — (PDF) [file pone.0309933.s005.pdf]

S4 Table. Associations between outdoor walk group attendance and the odds of experiencing improvement on health outcome measures from baseline to 3 months (without the Heckman correction)

	Comparisons between OWG attendance tertile groups		
	2nd tertile (10–15 sessions) vs 1st tertile (0–9 sessions)	3rd tertile (16–20 sessions) vs 1st tertile (0–9 sessions)	3rd tertile (16–20 sessions) vs 2nd tertile (10–15 sessions)
Measures	Odds ratio [95% CI]		
6-minute walk test	3.30 [0.34, 17.23]	6.16 [0.51, 83.35]	1.86 [0.34, 10.11]
10-meter walk test at comfortable pace	1.92 [0.47, 8.09]	3.19 [0.63, 17.62]	1.66 [0.49, 5.62]
10-meter walk test at fast pace	2.85 [0.70, 12.32]	8.41 [1.52, 56.28] *	2.96 [0.76, 11.41]
Mini-BESTest	0.65 [0.16, 2.54]	0.91 [0.19, 4.36]	1.40 [0.46, 4.30]
30-second sit-to-stand	0.43 [0.10, 1.65]	1.04 [0.20, 5.31]	2.43 [0.77, 7.71]
ASCQ	3.72 [0.91, 16.37] †	2.83 [0.56, 15.47]	0.76 [0.23, 2.54]
RAND-36 emotional well-being	0.60 [0.15, 2.39]	0.74 [0.15, 3.59]	1.23 [0.39, 3.87]

Notes: The odds ratios are in reference to the lower tertile/attendance group, with values greater than 1 suggesting potential dose-response relationships. All regression models were adjusted for participants' sex and study site. †p<.10; *p<.05
