# Supplementary material for: Examining dose-response of an outdoor walk group program in the Getting Older Adults Outdoors (GO-OUT) trial
Source: PLoS One. 2025 Mar 13;20(3):e0309933. doi: 10.1371/journal.pone.0309933 (PMC11906069; doi:10.1371/journal.pone.0309933)
Supplement: S5 Table — (PDF) [file pone.0309933.s006.pdf]

S5 Table. Associations between outdoor walk group attendance and total count of health outcomes with improved scores from baseline to 3 months (without the Heckman correction)

Outdoor walking attendance	Total count of improved health outcomes (range: 0–7)	
	Mean ± SD	Incident rate ratio [95% CI]
1st tertile (0–9 sessions)	4.00 ± 1.46	*1.00 (Reference)*
2nd tertile (10–15 sessions)	4.03 ± 1.69	1.07 [0.77, 1.48]
3rd tertile (16–20 sessions)	4.43 ± 1.50	1.22 [0.93, 1.76]
	Comparisons between 2nd and 3rd tertiles	
2nd tertile (10–15 sessions)		*1.00 (Reference)*
3rd tertile (16–20 sessions)		1.14 [0.88, 1.49]

Note: The incident rate ratios are in reference to the lower tertile/attendance group, with values greater than 1 suggesting potential dose-response relationships. The Poisson regression model was adjusted for participants' sex and study site.
